# ATP Synthase Inhibitory Factor-1 Deficiency Attenuates Doxorubicin Cardiotoxicity by Preserving Mitochondrial Structure and Function

**DOI:** 10.3390/ijms27146360

**Published:** 2026-07-17

**Authors:** Parnia Mobasheran, Ankit Aryal, Jazmine Aguilar, Scott Jennings, Lothar Lauterboeck, Kati Young, Qinglin Yang

**Affiliations:** 1 Department of Pharmacology and Experimental Therapeutics, School of Graduate Studies, Louisiana State University Health Sciences Center, New Orleans, LA 70112, USA; pmobas@lsuhsc.edu (P.M.);; 2 Cardiovascular Center of Excellence, Louisiana State University Health Science Center, New Orleans, LA 70112, USA; 3 School of Medicine, Louisiana State University Health Sciences Center, New Orleans, LA 70112, USA

**Keywords:** Doxorubicin, ATP synthase inhibitory factor 1 (IF1), mitochondrial dysfunction, cardiotoxicity

## Abstract

Doxorubicin (DOX) remains an effective chemotherapeutic agent, but its clinical use is limited by dose-dependent cardiotoxicity. Mitochondrial dysfunction and metabolic remodeling are central features of DOX-induced cardiac injury. ATP synthase inhibitory factor-1 (IF1) is an endogenous inhibitor of the hydrolytic activity of mitochondrial ATP synthase and has emerged as an important regulator of cellular bioenergetics. Cardiac IF1 expression is increased in multiple pathological conditions; however, its role in chemotherapy-induced cardiotoxicity remains unclear. Here, we investigated the contribution of IF1 to DOX-induced cardiotoxicity using male C57BL/6J wild-type (WT) and IF1 knockout (IF1KO) mice, isolated cardiac mitochondria, cultured neonatal cardiomyocytes, and AC16 human cardiomyocytes. Cardiac function was assessed by echocardiography, mitochondrial function by high-resolution respirometry and Seahorse metabolic flux analysis, and myocardial injury by histological and ultrastructural analyses. DOX treatment markedly increased cardiac IF1 protein levels despite reduced IF1 mRNA expression. IF1 deficiency enhanced mitochondrial respiration in isolated cardiac mitochondria and cultured cardiomyocytes under both basal and DOX-stressed conditions. IF1KO mice exhibited attenuated cardiac dysfunction and improved myocardial ultrastructure following DOX treatment compared with WT mice. In AC16 cardiomyocytes exposed to DOX, overexpression of WT IF1 improved cellular metabolic activity but provided only limited preservation of mitochondrial respiratory capacity. In contrast, overexpression of the dominant-negative IF1 mutant (IF1E30A) not only improved metabolic activity but also preserved mitochondrial respiration. These findings identify IF1 as a key regulator of metabolic adaptation during DOX stress. Upregulation of functional IF1 may represent an adaptive response that promotes glycolytic ATP production during mitochondrial stress, whereas inhibition of IF1 activity preserves metabolic activity primarily through maintenance of mitochondrial function. Collectively, these findings provide new insights into the role of IF1 in DOX-induced cardiomyopathy and highlight IF1 as a potential therapeutic target in cardio-oncology.

## 1. Introduction

Doxorubicin (DOX) is a widely used chemotherapeutic agent for the treatment of various malignancies; however, its clinical use is limited by cumulative, dose-dependent cardiotoxicity [1,2]. Recent studies suggest that up to 48% of patients receiving DOX may eventually develop heart failure [3]. Despite significant advances in understanding the mechanisms underlying DOX-induced cardiac injury, effective therapeutic strategies remain limited [4].

Mitochondria are a primary target of DOX and the main source of cellular ATP production. ATP synthase, located in the inner mitochondrial membrane, is a rotary enzyme complex driven by the proton motive force (PMF) to generate ATP. Under pathological stress, the collapse of the PMF can reverse ATP synthase activity, leading to ATP hydrolysis and proton pumping into the intermembrane space to maintain mitochondrial membrane potential (ΔΨm) at the expense of cellular ATP stores [5,6,7,8]. In parallel, cellular stress can induce mitochondrial matrix acidification, promoting the binding of ATP synthase inhibitory factor-1 (IF1) to ATP synthase. This interaction suppresses ATP hydrolysis and helps preserve intracellular ATP levels [9,10]. Thus, IF1 upregulation has been reported to confer protection in neurons and the heart [11,12,13]. In addition, IF1 overexpression has been linked to a metabolic reprogramming that supports anabolic pathways and rapid proliferation in many cancer cell lines [9,10].

Conversely, recent studies suggest that IF1 deficiency may attenuate maladaptive metabolic remodeling from oxidative phosphorylation (OXPHOS) to glycolysis, a hallmark of cardiac hypertrophy [12]. Additionally, inhibition of IF1 has been reported to ameliorate antimycin-induced mitochondrial toxicity [14]. Consistent with these findings, we previously demonstrated that IF1 knockout (IF1KO) mice exhibit enhanced resistance to pressure overload- and isoproterenol-induced cardiac stress [15].

Nevertheless, whether IF1 plays a critical role in regulating mitochondrial and cardiac function during DOX-induced cardiotoxicity remains unclear. In the present study, we hypothesized that IF1 modulates mitochondrial adaptation to DOX-induced stress and thereby influences cardiac susceptibility to DOX cardiotoxicity. To test this hypothesis, we investigated the effects of genetic IF1 deletion and overexpression on mitochondrial and cardiac function under DOX-induced stress.

## 2. Result

### 2.1. IF1 Deficiency Attenuates Dox-Induced Cardiac Dysfunction

**Figure 1:**
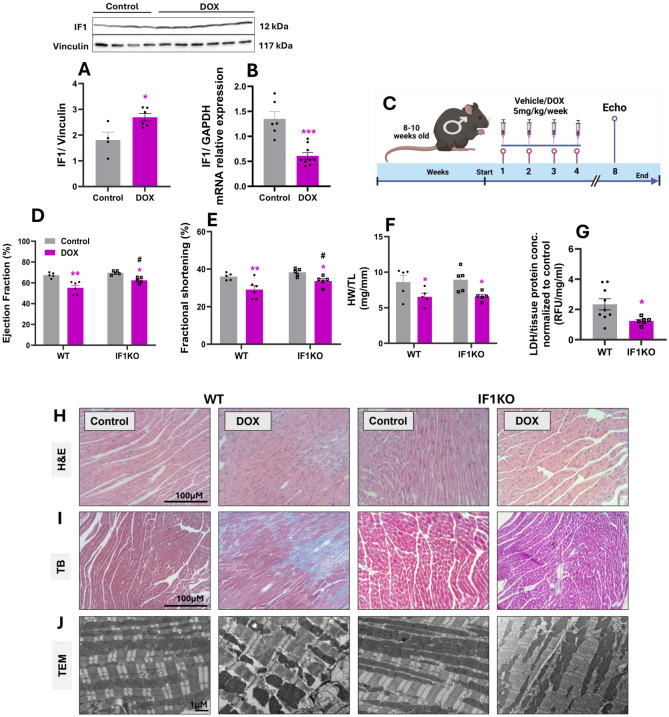
DOX induces upregulation of IF1 protein expression and silencing IF1 improves cardiac function in DOX-treated mice. (**A**) Representative Western blot images of IF1 protein levels across treatment groups and quantification of IF1 protein expression normalized to loading controls. (**B**) Relative mRNA expression levels of IF1 assessed by qPCR. (**C**) Schematic representation of experimental design using WT and IF1KO mice. Created in BioRender Mobasheran, P. (2026). https://BioRender.com/wjmmmgb (accessed on 2 June 2026) [1]. (**D**,**E**) Echocardiographic assessment of cardiac function, including ejection fraction (EF) and fractional shortening (FS). (**F**) heart weight to tibial length (HW/TL). (**G**) LDH level normalized to tissue protein. (**H**) Hematoxylin and Eosin (H&E) staining. (**I**) Trichrome blue (TB) staining. (**J**) Transmission electron microscopy (TEM). Panels A, B, and G were analyzed using Student’s *t*-test. Panels D–F were analyzed using two-way ANOVA followed by Fisher’s LSD post hoc test. (*) indicates comparisons between treated and control groups, * *p* < 0.05; ** *p* < 0.01, *** *p* < 0.0001. (#) indicates comparisons between WT and IF1KO genotypes, # *p* < 0.05. Circles indicate WT and squares indicate IF1KO mice.

To evaluate the impact of DOX on IF1 expression, we measured both protein and mRNA levels in mouse hearts. DOX treatment significantly increased cardiac IF1 protein expression (Figure 1A), whereas IF1 mRNA levels were significantly decreased compared with vehicle-treated controls (Figure 1B). We next assessed the role of IF1 in DOX-induced cardiotoxicity using WT and IF1KO mice, as outlined in Figure 1C. Echocardiographic analysis revealed that DOX induced a significant decline in ejection fraction (EF) and fractional shortening (FS) in both groups; however, this reduction was markedly attenuated in IF1KO mice (ΔEF = −10.4%) compared with WT mice (ΔEF = −18%) (Figure 1D,E). Heart weight normalized to tibial length (HW/TL) did not differ significantly between DOX-treated WT and IF1KO mice (Figure 1F). Cardiac LDH levels were significantly lower in DOX-treated IF1KO mice compared with DOX-treated WT mice after normalization to their respective untreated controls (Figure 1G). Histological assessment using H&E and Masson’s trichrome staining demonstrated better preservation of myocardial architecture and significantly reduced interstitial fibrosis in DOX-treated IF1KO hearts relative to WT hearts (Figure 1H,I). Consistent with these findings, representative transmission electron microscopy images qualitatively demonstrated preservation of sarcomeric organization and mitochondrial ultrastructure in DOX-exposed IF1KO hearts compared with WT controls (Figure 1J).

### 2.2. Silencing IF1 Enhances Mitochondrial Respiration at Baseline and Under Dox Stress

**Figure 2:**
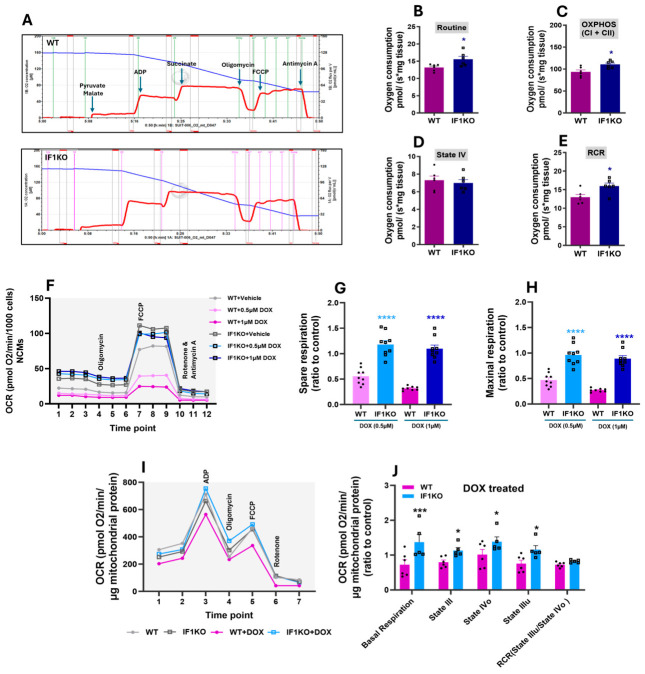
IF1 deletion improves mitochondrial respiration at baseline and under stress (**A**) Representative trace from the Oroboros O2k respirometer showing mitochondrial oxygen consumption (red line) and oxygen concentration in the chamber (blue line). (**B**–**E**) Quantification of mitochondrial respiratory parameters in isolated heart mitochondria from WT and IF1KO mice, including routine respiration, complex I- and II-linked respiration, state IV respiration (leak respiration), and respiratory control ratio (RCR; ratio of state III to state IV respiration) (**F**–**H**) Seahorse XF traces showing mitochondrial respiration in neonatal cardiomyocytes isolated from WT and IF1KO and quantification of maximal and spare respiratory capacity normalized to the respective control groups. (**I**,**J**) Mitochondrial respiration trace in isolated mitochondria measured using the Seahorse XFe24 analyzer and quantification of basal respiration, state III, state IV, state III, and RCR in mitochondria from DOX-treated WT and IF1KO mice, normalized to their respective control groups. Panels B–E were analyzed using Student’s *t*-test. Panels G and H were analyzed using one-way ANOVA followed by Tukey’s post hoc test. Panel J was analyzed using two-way ANOVA followed by Fisher’s LSD post hoc test. (*) indicates comparisons between WT and IF1KO, * *p* < 0.05, *** *p* < 0.001, **** *p* < 0.0001. Circles indicate WT and squares indicate IF1KO mice.

To determine the role of IF1 in regulating mitochondrial respiration, high-resolution respirometry was performed on isolated cardiac mitochondria from WT and IF1KO mice using the Oroboros O2k system (Figure 2A). Under basal conditions, IF1KO mitochondria exhibited significantly higher routine respiration compared with WT mitochondria. ADP-stimulated (State III) respiration supported by combined complex I and II substrates was also markedly increased in IF1KO mitochondria. In contrast, oligomycin-induced leak respiration (State IV) was comparable between genotypes. As a result, the respiratory control ratio (RCR) was significantly elevated in IF1KO mitochondria, indicating improved coupling efficiency (Figure 2B–E).

We next assessed mitochondrial function in neonatal cardiomyocytes (NCMs) isolated from WT and IF1KO mice using Seahorse XF analysis. Consistent with findings in isolated mitochondria, untreated IF1KO NCMs displayed significantly higher basal respiration, maximal respiration, and spare respiratory capacity than WT NCMs (Supplementary Figure S1A–D). Following DOX exposure (0.5 µM and 1 µM for 24 h), maximal respiration and spare respiratory capacity (normalized to untreated controls) were markedly better preserved in IF1KO NCMs relative to WT NCMs (Figure 2G,H).

These effects were corroborated in vivo by isolating cardiac mitochondria from DOX/vehicle-treated mice. Mitochondria from IF1KO hearts exhibited significantly higher basal respiration, State III (ADP-stimulated), State IV (leak), and uncoupled (State IIIu) respiration compared with those from WT hearts. However, the respiratory control ratio (RCR) did not differ significantly between groups (Figure 2I,J).

### 2.3. IF1 Modulation Differentially Affects Mitochondrial Respiration in Response to Substrate Availability

To further dissect the functional consequences of IF1 activity under metabolic stress, we next utilized gain-of-function and dominant-negative IF1 constructs in human AC16 cardiomyocytes. AC16 cells were transduced with adenoviral vectors expressing wild-type IF1 (IF1OE), the dominant-negative mutant IF1E30A, or GFP as a control. Western blot analysis confirmed robust overexpression of IF1 protein in both IF1OE- and IF1E30A-transduced cells compared with GFP controls (Supplementary Figure S2A). Parallel experiments were performed in glucose (+ glucose)- or galactose (+ galactose)-supplemented medium. Replacing glucose with galactose forces cells to rely primarily on oxidative phosphorylation for ATP production, thereby unmasking potential mitochondrial defects that may be masked by glycolytic compensation [16].

In + glucose medium under untreated conditions, both IF1OE and IF1E30A significantly reduced maximal respiration and spare respiratory capacity compared with GFP-transduced control cells (Supplementary Figure S2B,I). Following 24 h exposure to 1 µM DOX, IF1OE-transduced cells showed no significant change in basal respiration but exhibited elevated maximal and spare respiratory capacity relative to controls (Supplementary Figure S2C,D). IF1E30A displayed more pronounced protection, with significant increases in basal, maximal, and spare respiratory capacity (Supplementary Figure S2J,K). Notably, both IF1OE and IF1E30A cells showed increased ATP production following DOX treatment, with no significant changes in proton leak. 

**Figure 3:**
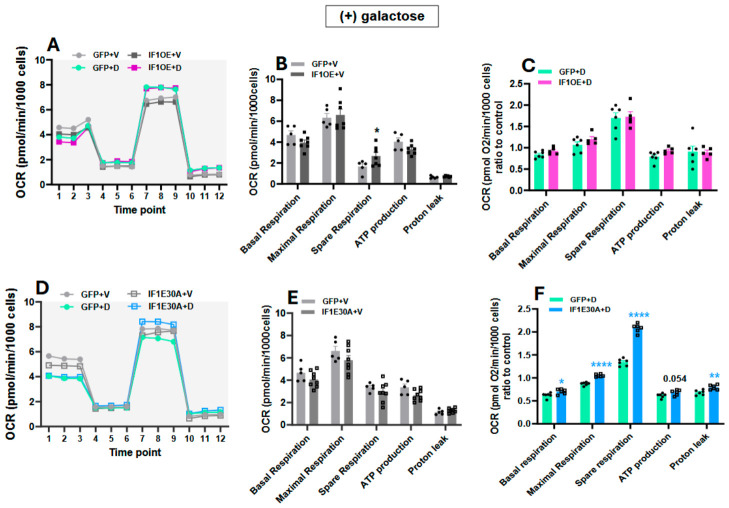
IF1OE fails to sustain mitochondrial respiration under stress in galactose-driven media, whereas IF1E30A preserves mitochondrial respiration. (**A**) Representative Seahorse XF Mito Stress Test trace showing OCR profiles of IF1OE and WT cells cultured in galactose-containing media. (**B**) Quantification of mitochondrial parameters including basal respiration, maximal respiration, spare respiratory capacity, ATP-linked respiration, and proton leak under steady-state conditions. (**C**) Mitochondrial respiratory parameters following DOX exposure, normalized to their respective steady-state values. (**D**) Representative Seahorse XF Mito Stress Test trace showing OCR profiles of IF1E30A and WT cells under basal conditions. (**E**) Quantification of mitochondrial parameters including basal respiration, maximal respiration, spare respiratory capacity, ATP-linked respiration, and proton leak under steady-state conditions. (**F**) Mitochondrial parameters following DOX exposure, normalized to their respective steady-state values. Data are presented as mean ± SEM, and statistical significance was determined using the two-way ANOVA following by Fisher’s LSD test. (*) indicates comparisons between GFP and IF1OE or IF1E30A groups, * *p*  <  0.05; ** *p*  <  0.01; **** *p*  <  0.000. Circles indicate GFP and squares indicate IF1OE or IF1E30A transduced cells.

In + galactose, under untreated conditions, the reductions in respiratory parameters previously observed in glucose-containing medium were no longer apparent (Figure 3B,E), indicating that the mitochondria in both IF1OE- and IF1E30A are intrinsically functional. Following DOX exposure, IF1OE cells failed to recapitulate the protective respiratory phenotype observed in glucose medium; only ATP-linked respiration remained significantly elevated (Figure 3C). In contrast, IF1E30A consistently exhibited higher basal, maximal, and spare respiratory capacities, accompanied by increased proton leak and a trend toward elevated ATP production (Figure 3F).

### 2.4. IF1 Modulation Promotes Distinct Metabolic Profiles Under Dox Stress

**Figure 4:**
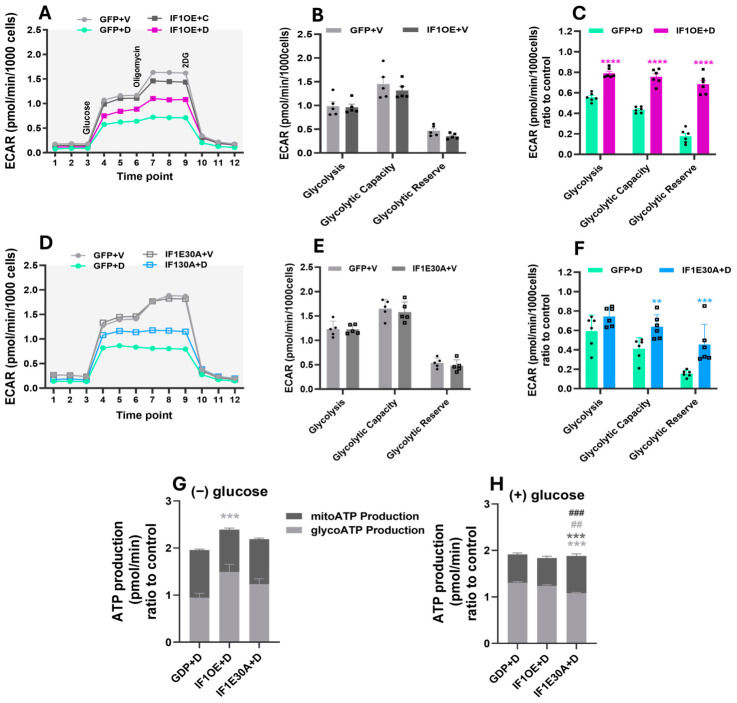
IF1E30A exhibited distinct regulation of glycolytic capacity and mitochondrial membrane potential compared with IF1KO and control cells. (**A**) ECAR trace of IF1OE and WT cells measured using the Seahorse XF Glycolysis Stress Test. (**B**) Quantification of glycolytic parameters, including basal glycolysis, glycolytic capacity, and glycolytic reserve, under steady-state conditions (**C**) Glycolysis parameters following DOX exposure, normalized to the respective steady-state values (**D**) ECAR trace of IF1E30A and WT cells measured under the same assay conditions. (**E**) Quantification of glycolytic parameters under steady-state conditions. (**F**) Normalized glycolytic parameters after DOX treatment. (**G**,**H**) Relative contribution of mitochondrial ATP production (mitoATP) and glycolytic ATP production (glycoATP) to total ATP production measured using the Seahorse XF Real-Time ATP Rate Assay. For panels (**A**–**F**), statistical significance was determined using two-way ANOVA followed by Fisher’s LSD post hoc test. For panels (**G**) and (**H**), statistical significance was determined using two-way ANOVA followed by Tukey’s post hoc test. (*) indicates comparisons between GFP control and IF1OE or IF1E30A groups. ** *p* < 0.01, *** *p* < 0.001, **** *p* < 0.0001. (#) indicates comparisons between IF1OE and IF1E30A groups; light gray represents glycolytic ATP, and dark gray represents mitochondrial ATP production. ## *p* < 0.01, ### *p* < 0.001. Circles indicate GFP and squares indicate IF1OE or IF1E30A transduced cells.

To determine whether IF1 modulation alters glycolytic reliance under DOX stress, glycolytic function was assessed using the Seahorse Glycolysis Stress Test. Under untreated conditions, no significant differences were observed in glycolytic rate, glycolytic capacity, or glycolytic reserve among GFP, IF1OE-, and IF1E30A cells (Figure 4A,B,D,E).

Following DOX exposure, however, IF1OE exhibited a marked increase in glycolytic rate, glycolytic capacity, and glycolytic reserve compared with GFP controls (Figure 4C), indicating a pronounced shift toward glycolytic metabolism under stress. In contrast, IF1E30A showed only modest increases in glycolytic capacity and reserve, with no significant change in basal glycolytic rate relative to controls (Figure 4F).

We next quantified the relative contributions of glycolysis and oxidative phosphorylation to total ATP production using the Seahorse XF Real-Time ATP Rate Assay. In glucose- and galactose-free medium (− glucose), IF1OE exhibited a significantly higher basal glycoATP production rate compared with GFP and IF1E30A cells (Figure 4G), while mitoATP production rates were comparable across all three groups. Upon addition of glucose, GFP and IF1OE cells displayed similar levels of both glycoATP and mitoATP production. Notably, IF1E30A exhibited a significantly lower glycoATP production and higher mitoATP production relative to both GFP and IF1OE (Figure 4H).

### 2.5. Distinct Regulation of Mitochondrial Membrane Profile and Cellular Metabolic Activity by IF1 Variants

**Figure 5:**
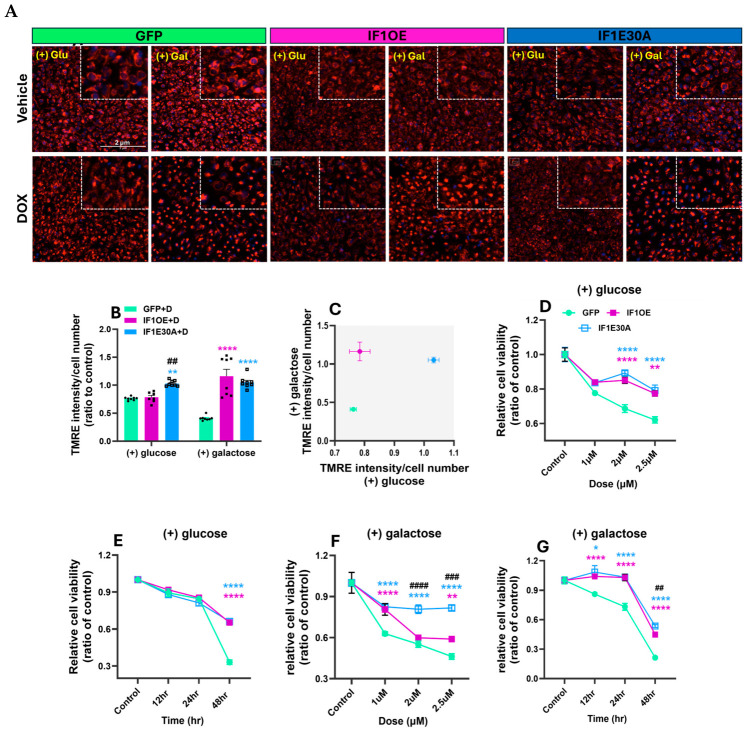
IF1 modulation exhibited distinct stress-response phenotypes under glucose- and galactose-containing conditions. (**A**) TMRE and DAPI staining images of AC16 cells transduced with IF1OE or IF1E30A, compared to WT, under (+) glucose and (+) galactose conditions. (**B**) Quantification of TMRE fluorescence intensity normalized to the untreated respective experimental group. (**C**) Correlation between fluorescence intensity measured in (+) glucose and (+) galactose media in GFP, IF1OE, and IF1E30A cells. (**D**,**E**) Dose-dependent and time-dependent effects of IF1OE and IF1E30A under DOX-induced stress, normalized to their respective untreated experimental groups in (+) glucose medium. (**F**,**G**) Dose-dependent and time-dependent effects of IF1OE and IF1E30A under DOX-induced stress, normalized to their respective untreated experimental groups in (+) galactose medium. For panels (**B**,**C**), statistical significance was determined using two-way ANOVA followed by Fisher’s LSD post hoc test. For panels (**D**,**G**), statistical significance was determined using two-way ANOVA followed by Tukey’s post hoc test. (*) indicates comparisons between GFP-transduced control cells and the other groups. * *p* < 0.05, ** *p* < 0.01, **** *p* < 0.0001. (#) indicates comparisons between IF1OE and IF1E30A groups. ## *p* < 0.01, ### *p* < 0.001, #### *p* < 0.001. Circles indicate GFP, filled squares indicate IF1OE, and open squares indicate IF1E30A-transduced cells.

To assess the impact of IF1 expression on ΔΨm, TMRE staining was performed in AC16 cardiomyocytes. Under steady-state conditions, GFP cells exhibited significantly higher ΔΨm compared with both IF1OE and IF1E30A, irrespective of whether cells were cultured in glucose- or galactose-supplemented medium (Figure 5).

Following DOX exposure in (+) glucose medium (normalized to respective untreated controls), IF1OE showed no significant change in ΔΨm relative to GFP control cells. In contrast, IF1E30A-transduced cells displayed a significant increase in ΔΨm, compared to both IF1OE and GFP (Figure 5B). In (+) galactose media, IF1OE exhibited a significant elevation in ΔΨm compared with GFP controls, whereas IF1E30A maintained their already elevated ΔΨm with no further increase (Figure 5B). The relationship between culture medium (glucose vs. galactose) and ΔΨm across the three groups under DOX stress is summarized in Figure 5C.

Cellular metabolic activity was assessed using the MTT assay. In (+) glucose, no significant differences were observed among groups after 24 h exposure to 1 µM DOX. However, at higher DOX concentrations (2 and 2.5 µM), both IF1OE and IF1E30A showed significantly higher metabolic activity than GFP controls (Figure 5D). Time-course analysis with 1 µM DOX further revealed that both IF1OE and IF1E30A preserved metabolic activity significantly better than controls at 12, 24, and 48 h post-exposure (Figure 5E). In (+) galactose media, both IF1OE and IF1E30A cells displayed significantly higher activity than GFP controls at 1 µM DOX. At higher doses, IF1E30A showed superior preservation compared with IF1OE (Figure 5F). In time-dependent experiments, IF1E30A conferred significantly higher metabolic activity than both IF1OE and GFP cells at 48 h (Figure 5G). However, after 48 h exposure to 1 μM DOX, the ratio of dead cells to total cells, normalized to the corresponding untreated control groups, showed a trend toward reduced cell death in IF1OE cells compared with GFP cells in glucose media. In galactose media, IF1E30A cells exhibited lower cell death than GFP cells and a trend toward reduced cell death compared with IF1OE cells (Supplementary Figure S3A,B).

## 3. Discussion

DOX-induced cardiotoxicity is characterized by profound mitochondrial dysfunction and impaired bioenergetic homeostasis [17]. In the present study, we identify IF1 as an important modulator of mitochondrial function under DOX-induced stress. Our data demonstrate that DOX treatment increases cardiac IF1 protein levels despite reduced transcript abundance, suggesting post-transcriptional regulation. Functionally, genetic ablation of IF1 preserves mitochondrial respiratory capacity and improves cardiac structure and function following DOX exposure, supporting a maladaptive role of IF1 in this setting.

A notable finding of this study is the dissociation between IF1 expression and its functional consequences. While IF1 deficiency consistently improved mitochondrial respiration and cardiac outcomes, IF1 overexpression did not uniformly exacerbate mitochondrial dysfunction. This apparent discrepancy suggests that IF1 activity is not solely determined by its abundance but is highly dependent on mitochondrial energetic state. IF1 is known to bind and inhibit the ATP synthase preferentially under conditions of mitochondrial depolarization and matrix acidification [18,19]. Therefore, under basal or mildly stressed conditions, increased IF1 expression may have limited functional effects, whereas under mitochondrial stress, IF1 becomes functionally engaged, influencing ATP synthase activity and, further, metabolic reprogramming [12]. One possible interpretation is that the increased IF1 protein abundance following DOX exposure represents an adaptive response to mitochondrial stress, as suggested by previous studies [20,21,22]. Whether sustained IF1-mediated inhibition of ATP synthase becomes maladaptive during chronic DOX exposure remains to be determined, as temporal studies were not performed in the present investigation. In DOX-treated mice, cardiac IF1 protein increased despite declining mRNA compared with vehicle-treated mice, suggesting post-transcriptional regulation, possibly through enhanced protein stability or stress-induced translational control [23,24,25].

The in vivo role of IF1 has been investigated in several cardiovascular settings. Activation of GPR35 has been reported to promote IF1-ATP synthase interaction, inhibiting ATP hydrolysis, reducing ROS production, and preventing mitochondrial permeability transition pore (mPTP) formation [13]. IF1-induced ROS signaling has also been suggested to activate the AMPK pathway, improving cardiac function through maintenance of energy homeostasis [26]. Conversely, recent studies show that cardiac-specific deletion of IF1 prevents metabolic remodeling and protects against pathological cardiac remodeling during chronic stress [12]. This finding is also consistent with our earlier finding that IF1KO mice are more resistant to pressure overload and isoproterenol-induced cardiac stress [15]. In the present study, deletion of IF1 similarly alleviated DOX-induced cardiac dysfunction and histological injury.

The ATP synthase inhibitory role of IF1 has been proposed as a short-term adaptive mechanism during energetic stress. Increased ATP synthase–IF1 interaction has been linked to the formation of nonproductive FoF1-ATP synthase oligomers, HIF-1α activation, and metabolic reprogramming from OXPHOS toward glycolysis [12,27]. Consistent with this model, mitochondria-enriched preparations and neonatal cardiomyocytes from IF1KO mice exhibited higher mitochondrial respiration under both basal and DOX-stressed conditions.

In AC16 cardiomyocytes transduced with adenoviral vectors expressing either WT IF1 (IF1OE) or the dominant-negative mutant IF1E30A [14], both IF1OE and E30A reduced respiration under basal conditions [27,28]. However, IF1E30A and, to a lesser extent, IF1OE preserved mitochondrial respiration under DOX stress. This dual IF1 behavior is consistent with prior studies showing that both IF1 silencing [12,14,27] and overexpression [26,29] can be protective under different pathological contexts, likely through distinct downstream signaling pathways. IF1OE has been shown to promote a transient metabolic shift from OXPHOS toward glycolysis [12,30,31], and our findings similarly demonstrated enhanced glycolytic activity in IF1OE cells under DOX stress. Although IF1E30A cells also exhibited increased glycolytic capacity and reserve, the magnitude of this effect was less pronounced than in IF1OE cells. Direct assessment of glycolytic flux will be important to further define the metabolic adaptations associated with IF1 modulation.

Under glucose-deprived conditions with pyruvate and glutamine as the primary metabolic substrates, IF1OE cells exhibited higher estimated glycolytic ATP (glycoATP) production rates without significant changes in mitochondrial ATP (mitoATP) production compared with control cells. This difference was no longer observed under glucose-replete conditions. In contrast, IF1E30A cells displayed higher estimated mitoATP production rates accompanied by lower glycoATP production under glucose-containing conditions, indicating a greater reliance on mitochondrial oxidative metabolism. These findings suggest that wild-type IF1 overexpression and expression of the dominant-negative IF1E30A mutant differentially influence cellular energy metabolism in a substrate-dependent manner. However, because ATP production rates were estimated from Seahorse metabolic flux measurements rather than direct biochemical assays, future studies measuring ATP synthase activity and intracellular ATP content will be required to define the underlying mechanisms more precisely.

Mitochondrial respiration increased markedly when cells were cultured in galactose rather than glucose, reaching levels comparable to controls in both IF1OE and IF1E30A cells. These findings indicate that the suppressed respiration observed under glucose conditions reflects substrate-dependent metabolic adaptation rather than intrinsic mitochondrial dysfunction [32]. Under DOX-induced stress, IF1E30A cells exhibited significantly greater basal, maximal, and spare respiratory capacities, indicating that inhibition of functional IF1 preserves mitochondrial flexibility and respiratory reserve when glycolytic compensation is limited [32]. Consistent with these findings, IF1E30A cells maintained ΔΨm more effectively than both control and IF1OE cells under stress conditions. Previous studies demonstrated that maintenance of ΔΨm during energetic stress partially depends on ATP hydrolysis and reverse ATP synthase activity [20,33]. In galactose-containing medium, all groups exhibited higher ΔΨm than in glucose-containing medium, consistent with greater OXPHOS dependency and reduced reliance on ATP hydrolysis to maintain ΔΨm [4,16,34]. Whether IF1 directly regulates ATP synthase assembly, mitochondrial dynamics, or cristae remodeling during DOX injury remains unknown.

In galactose-containing medium, where cells rely predominantly on mitochondrial oxidative phosphorylation for ATP production, the MTT assay, which primarily reflects cellular reductive capacity and mitochondrial metabolic activity, demonstrated that IF1E30A cells exhibited greater resistance to both prolonged low-dose DOX exposure compared with IF1OE and control cells. Consistent with these findings, direct assessment of cell death using SYTOX™ Deep Red staining showed a lower ratio of dead cells to total cells in IF1E30A cultures following prolonged 1μM DOX exposure. Together, these findings support the concept that inhibition of functional IF1 preserves mitochondrial function and promotes cell survival during DOX exposure particularly when glycolytic compensation is absent.

In conclusion, our findings identify IF1 as a context-dependent regulator of mitochondrial bioenergetics in DOX-induced cardiotoxicity and highlight IF1 as a potential therapeutic target for mitigating chemotherapy-induced cardiotoxicity.

Emerging evidence suggests that ATP synthase plays a broader role beyond ATP production, including the regulation of ΔΨm, cristae structure, and mitochondrial dynamics [14,30,31,35,36]. Because the present study employed a global IF1 KO model, the relative contributions of cardiomyocyte-autonomous and systemic effects remain to be determined. Furthermore, IF1 is overexpressed in many tumor types and has been associated with enhanced proliferative growth [37,38,39]. Further studies should determine whether systemic IF1 inhibition can simultaneously attenuate DOX-induced cardiotoxicity while preserving, or potentially enhancing, anticancer efficacy.

## 4. Materials and Methods

### 4.1. Drugs and Reagents

Doxorubicin (DOX), D-galactose, carbonyl cyanide 4-(trifluoromethoxy)phenylhydrazone (FCCP), oligomycin, rotenone, antimycin A, pyruvate, malate, glutamate, succinate, 2-deoxy-D-glucose, and rat tail collagen type I were obtained from Sigma-Aldrich (St. Louis, MO, USA), unless otherwise specified. Tetramethylrhodamine ethyl ester (TMRE; Cat. No. T669), SYTOX™ Deep Red Nucleic Acid Stain, Hoechst 33342, RIPA buffer, protease and phosphatase inhibitor cocktails, the Pierce™ BCA Protein Assay Kit, the Pierce™ Primary Cardiomyocyte Isolation Kit, and the Power SYBR™ Green RNA-to-CT™ 1-Step Kit were purchased from Thermo Fisher Scientific (Waltham, MA, USA). Dulbecco’s modified Eagle medium/F-12 (DMEM/F-12), fetal bovine serum, penicillin–streptomycin, and other routine cell-culture reagents were obtained from Gibco, Thermo Fisher Scientific (Waltham, MA, USA). Seahorse XF assay media, calibrant, XF24 cell culture microplates, and the Seahorse XF Cell Mito Stress Test, Glycolysis Stress Test, and Real-Time ATP Rate Assay reagents were obtained from Agilent Technologies (Santa Clara, CA, USA). The miRNeasy Mini Kit was purchased from QIAGEN (Hilden, Germany). The lactate dehydrogenase assay kit was obtained from Abcam (Cat. No. ab197000; Cambridge, UK). Western blotting reagents, including 4× Laemmli loading buffer, 4–15% TGX Stain-Free gels, PVDF membranes, and enhanced chemiluminescence reagents, were obtained from Bio-Rad Laboratories (Hercules, CA, USA). Primary antibodies against IF1 (Abcam, Cat. No. ab110277; 1:500; Cambridge, UK) and vinculin (Santa Cruz Biotechnology, Cat. No. sc-25336; 1:500; Dallas, TX, USA), as well as HRP-conjugated anti-rabbit IgG (Cell Signaling Technology, Cat. No. 7074; 1:1000; Danvers, MA, USA) and anti-mouse IgG (Cell Signaling Technology, Cat. No. 7076; 1:1000; Danvers, MA, USA), were used for immunoblotting. All other chemicals were of analytical grade.

### 4.2. Experimental Animals

All animal procedures were conducted in accordance with the National Institutes of Health Guide for the Care and Use of Laboratory Animals [2] and complied with the U.S. Department of Agriculture Animal Welfare Act [3]. Experimental protocols involving mice, including neonatal animals, were approved by the Institutional Animal Care and Use Committee (IACUC; protocol #4442 and 4209) at Louisiana State University Health Sciences Center (LSUHSC). Animals were housed in a temperature- and humidity-controlled environment under a 12 h light–dark cycle, with all efforts made to minimize animal distress. IF1KO (C57BL/6J) mice were originally obtained from Dr. Yoshida’s group (department of Molecular Bioscience, Kyoto Sangyo University, Kyoto, Japan) [15,30,40]. WT (C57BL/6J) mice were obtained from The Jackson Laboratory (Bar Harbor, ME, USA). IF1KO mice are viable, fertile, and show no baseline cardiac dysfunction [4].

### 4.3. Animal Models and In Vivo Treatment Protocol

Male WT (n = 5) and IF1KO (n = 6) mice aged 8–10 weeks were randomly assigned to vehicle or Doxorubicin treatment groups. Mice received weekly intraperitoneal (i.p.) injections of Doxorubicin (DOX; 5 mg/kg in saline, Sigma) for four consecutive weeks. Control animals received equivalent volumes of vehicle (saline). Cardiac function was assessed using echocardiography three weeks after the final DOX dose, after which mice were euthanized for terminal analyses.

### 4.4. Cardiac Functional and Structural Assessments

Echocardiography was conducted as previously described [41]. Before each echocardiography session, the operator concealed the animal identity. Mice were anesthetized with 5% isoflurane in medical oxygen (1 L/min) for induction and maintained at 1–2% isoflurane delivered via a nose cone while positioned supine on a heated stage. Heart and respiratory rates were continuously monitored using stage electrodes. Chest hair was removed with a depilatory cream (Nair, NJ, USA) to optimize image quality. Cardiac images were acquired using a VEVO F2 system (FUJIFILM VisualSonics Inc., Toronto, ON, Canada) equipped with a 22–55 MHz transducer, with heart rate maintained between 400 and 500 beats per minute. M-mode recordings were obtained, and the mean values from four consecutive cardiac cycles were used for systolic function analysis. All images were analyzed using Vevo LAB software (FUJIFILM VisualSonics Inc., Toronto, ON, Canada).

### 4.5. Histology

Hearts were perfused with phosphate-buffered saline (PBS) and then fixed with 4% paraformaldehyde (PFA). Fixed hearts were paraffin-embedded, longitudinally sectioned into 5-μm-thick slices, and stained with hematoxylin and eosin (H&E) or Masson’s trichrome (AML Laboratories) for histological evaluation. Fibrotic remodeling was assessed based on the extent and intensity of blue staining in Masson’s trichrome-stained sections. Images were acquired using an Olympus light microscope equipped with digital imaging software (Olympus Corporation, Tokyo, Japan), with a minimum of three representative fields captured per tissue section.

### 4.6. Transmission Electron Microscopy (TEM)

Heart tissue was excised and cut into approximately 1 mm^3^ pieces before fixation. Samples were processed by the Louisiana State University Electron Microscopy Core Facility (Baton Rouge, LA, USA). Tissue was fixed in Karnovsky’s fixative, post-fixed in 1% osmium tetroxide, stained en bloc with 1% uranyl acetate, dehydrated through graded ethanol solutions, and embedded in EponEmbed 812 resin using a BioWave microwave tissue processor (Ted Pella, Inc., Redding, CA, USA). Resin blocks were polymerized at 60 °C for 24–48 h. Ultrathin sections were prepared using a Leica EM UC7 ultramicrotome (Leica Microsystems, Wetzlar, Germany), mounted on carbon-coated copper grids, and counterstained with Reynolds lead citrate. Sections were examined using a JEOL JEM-1400 TEM (JEOL Ltd., Tokyo, Japan), and digital images were acquired with a Gatan digital camera (Gatan Inc., Pleasanton, CA, USA) by a technician blinded to sample identity. Cardiac samples from two animals per experimental group was evaluated. Representative images are presented as qualitative evidence of ultrastructural differences between groups.

### 4.7. Primary Neonatal Cardiomyocyte Isolation

Neonatal mouse cardiomyocytes (NCMs) were isolated from 1–3-day-old pups using the Pierce™ Primary Cardiomyocyte Isolation Kit (Thermo Fisher Scientific, Waltham, MA, USA) according to the manufacturer’s instructions. Hearts were excised, minced into small fragments, and enzymatically dissociated using the kit-supplied digestion enzymes. Following isolation, cell yield and viability were assessed by trypan blue exclusion using a hemocytometer. Cell viability typically ranged from 50 to 60 cells were plated and cultured in DMEM supplemented with 10% fetal bovine serum (FBS) and 1% penicillin–streptomycin for 24 h to allow cell attachment. The medium was then replaced with fresh medium containing the cardiomyocyte-specific growth supplement provided in the kit, which is designed to promote cardiomyocyte survival and reduce fibroblast proliferation. Cells were maintained at 37 °C in a humidified incubator with 5% CO_2_ and used for experiments after an additional 24 h in culture. Functional cardiomyocyte cultures were evidenced by spontaneous synchronous contractions, which were routinely observed within 24 h after addition of the cardiomyocyte growth supplement. Cell culture experiments were performed using at least three independent biological replicates unless otherwise stated.

### 4.8. AC16 Cell Culture and Adenoviral Transduction

AC16 human cardiomyocytes obtained from ATCC (ATCC, CRL-2921; Manassas, VA, USA) were seeded onto rat tail collagen type I-coated plates (Sigma; 1:40 dilution) and maintained in DMEM/F12 medium (Gibco, Thermo Fisher Scientific, Waltham, MA, USA) supplemented with 12.5% FBS and 1% penicillin–streptomycin. Cells were first transduced with adenoviral constructs encoding GFP-tagged IF1 or the dominant-negative mutant IF1E30A VectorBuilder Inc. (Chicago, IL, USA) at a multiplicity of infection (MOI) of 75, while GFP-transduced cells served as controls. Following transduction, cells were either maintained in glucose-containing medium that contained either 17.5 mM glucose or switched to galactose-supplemented medium containing 10 mM galactose (Sigma-Aldrich, Cat. No. G5388; St. Louis, MO, USA) and allowed to adapt for 24 h. Cells were then treated with DOX (Sigma-Aldrich, St. Louis, MO, USA; 1 μM) for an additional 24 h before harvest for subsequent in vitro analyses. Cell culture experiments were performed using at least three independent biological replicates unless otherwise specified. Depending on the assay, cells were seeded at a density of 12,000–15,000 cells per well.

### 4.9. Mitochondrial Membrane Potential (ΔΨm)

Mitochondrial membrane potential was evaluated using Tetramethylrhodamine ethyl ester (TMRE) (Thermo Fisher Scientific, Cat. No. T669; Waltham, MA, USA). Neonatal cardiomyocytes were incubated with 200 nM TMRE in phenol red-free culture medium at 37 °C for 30 min. Cells were then gently washed with pre-warmed PBS to and immediately imaged using Cytation™ 5 Cell Imaging Multi-Mode Reader (BioTek Instruments, Agilent Technologies, Winooski, VT, USA). As a positive control for mitochondrial depolarization, a subset of cells was pretreated with 100 µM carbonyl cyanide 4-(trifluoromethoxy) phenylhydrazone; (FCCP, Sigma-Aldrich) for 15 min prior to TMRE staining.

### 4.10. MTT Assay

Cellular metabolic activity was assessed using an MTT assay (Thermo Fisher Scientific, Waltham, MA, USA). AC16 cells were seeded at a density of 15,000 cells/well and maintained in either glucose- or galactose-containing DMEM media. Cells were transduced with the indicated adenoviral vectors for 24 h prior to DOX treatment. MTT was dissolved in sterile PBS, and cells were incubated with MTT-containing complete culture medium for 4 h at 37 °C in a humidified 5% CO_2_ incubator. Following incubation, the medium was removed, and the resulting formazan crystals were dissolved in DMSO. Background absorbance was determined using cell-free wells containing the same medium and MTT reagent, and this value was subtracted from all measurements. Absorbance was measured at 570 nm using a Varioskan LUX Multimode Microplate Reader (Thermo Fisher Scientific, Waltham, MA, USA). All experiments were performed using three independent biological replicates.

### 4.11. Cell Death Assay

AC16 cells were seeded at a density of 12,000 cells/well in 96-well plates and subjected to the indicated treatments. Following treatment, cells were incubated with 1 μM SYTOX™ Deep Red Nucleic Acid Stain and 3 μM Hoechst 33342 (Thermo Fisher Scientific, Waltham, MA, USA). for 15 min at 37 °C. Fluorescence images were acquired using a Cytation 5 Cell Imaging Multi-Mode Reader. Dead cells were identified as SYTOX-positive cells, while total cell number was determined by Hoechst-positive nuclei. Cell death was expressed as the ratio of SYTOX-positive cells to total Hoechst-positive cells and normalized to the corresponding vehicle-treated control groups.

### 4.12. Seahorse XF24 Stress Test on Cells

NCMs (80,000 cells/well) or AC16 cells (15,000 cells/well) were seeded into XF24 cell culture microplates (Agilent Technologies, Santa Clara, CA, USA) and treated with DOX for 24 h. Seahorse XF24 sensor cartridges were hydrated overnight in Seahorse XF Calibrant at 37 °C in a non-CO_2_ incubator. On the day of the assay, cells were washed and equilibrated in Seahorse XF assay DMEM medium without serum and phenol red, supplemented with 10 mM glucose or, when indicated, galactose, 2 mM L-glutamine, and 1 mM sodium pyruvate for 45–60 min prior to measurement. Oxygen consumption rate (OCR) was measured following sequential injections of oligomycin (1.5 μM), FCCP (3 μM), and rotenone/antimycin A (1 μM each) using the Seahorse XF Cell Mito Stress Test Kit (Agilent Technologies, Santa Clara, CA, USA).

For the glycolysis stress test, cells were incubated in Seahorse XF DMEM assay medium supplemented with 2 mM L-glutamine. Extracellular acidification rate (ECAR) was measured following sequential injections of glucose (10 mM), oligomycin (1 μM), and 2-deoxy-D-glucose (2-DG; 50 mM) using the Seahorse XF Cell Glycolysis Stress Test Kit (Agilent Technologies, Santa Clara, CA, USA).

For the Real-Time ATP Rate Assay, baseline OCR and ECAR were measured in glucose-free assay medium prior to injection of glucose (10 mM final concentration), followed by sequential injections of oligomycin (1 μM) and rotenone/antimycin A (0.5 μM each) to determine mitochondrial and glycolytic ATP production rates. For normalization, the final injection port contained Hoechst 33342 at a final concentration of 3 μM. Nuclei were quantified using a Cytation 5 Cell Imaging Multi-Mode Reader, and OCR/ECAR values were normalized to cell number for comparison across experimental groups.

### 4.13. Heart Mitochondrial Isolation

Enriched mitochondrial fractions were isolated from mouse hearts based on our previously published protocol [42]. Briefly, hearts were rapidly excised, cleared of blood and major vessels, and placed in ice-cold buffer A containing 250 mM sucrose, 10 mM Tris-HCl, and 0.5 mM EDTA (pH 7.4). Cardiac tissue was washed, minced into approximately 1 mm^3^ pieces, and homogenized in buffer A using a Potter–Elvehjem homogenizer (4–6 strokes). The homogenate was centrifuged at 1000× *g* for 10 min at 4 °C to remove tissue debris and nuclei. The resulting supernatant was subsequently centrifuged at 8000× *g* for 5 min to pellet mitochondria. The mitochondrial pellet was washed, centrifuged again, and resuspended in buffer A (100–150 μL per heart) and maintained on ice until use. Mitochondrial protein concentration was determined using a BCA protein assay kit (Thermo Fisher Scientific, Waltham, MA, USA).

### 4.14. Seahorse XF24 Mitochondrial Stress Test on Isolated Mitochondria

Enriched isolated mitochondria were diluted to a final concentration of 5 μg protein per 50 μL in mitochondrial assay solution (MAS) containing 220 mM D-mannitol, 70 mM sucrose, 10 mM KH_2_PO_4_, 5 mM MgCl_2_, 2 mM HEPES, 1 mM EGTA, and 0.2% (*w*/*v*) fatty acid-free BSA in ultrapure water, adjusted to pH 7.2 with KOH at 37 °C. On the day of the assay, MAS was supplemented with 10 mM pyruvate and 2 mM malate. A total of 50 μL of mitochondrial suspension was added to each well of a Seahorse XFe24 cell culture microplate and centrifuged at 2000× *g* for 20 min at 4 °C to facilitate mitochondrial attachment. Following centrifugation, 150 μL of pre-warmed MAS supplemented with pyruvate and malate was gently added to each well, and the plate was incubated at 37 °C in a non-CO_2_ incubator for 10 min prior to analysis. Injection ports of the Seahorse sensor cartridge were loaded with 10× concentration for the final concentration of ADP (5 mM), oligomycin (5 μM), FCCP (5 μM), antimycin A (10 μM), and rotenone (2 μM). Mitochondrial respiratory parameters were determined as follows: basal respiration, ADP-stimulated respiration (State III; OXPHOS capacity), oligomycin-induced leak respiration (State IV), and FCCP-stimulated maximal respiration (State IIIu). The respiratory control ratio (RCR) was calculated as the ratio of State III to State IV respiration.

### 4.15. High-Resolution Respirometry (OROBOROS O2K)

High-resolution mitochondrial respiration was assessed using the Oxygraph-2k system (Oroboros Instruments, Innsbruck, Austria) as previously described [20]. Prior to each experiment, chambers were rinsed with double-distilled water and filled with 2.5 mL MiR05 respiration buffer (Oroboros Instruments, Innsbruck, Austria) for air calibration. Following stabilization of the oxygen signal, freshly enriched isolated mitochondria (200 μg protein) were added to each chamber. Mitochondrial respiration was assessed using a substrate–uncoupler–inhibitor titration (SUIT) protocol. Pyruvate (5 mM), malate (5 mM), and glutamate (10 mM) were initially added to establish baseline respiration through complex I-linked substrates. ADP (1 mM) and succinate (10 mM) were subsequently introduced to assess complex I+II-linked oxidative phosphorylation (State III respiration). Oligomycin (2 μg/mL) was then added to determine LEAK respiration (State IV). Maximal electron transport system (ETS) capacity was evaluated by stepwise titration of FCCP (0.5 μM increments). Finally, antimycin A (2.5 μM) was added to inhibit complex III and determine non-mitochondrial oxygen consumption. The respiratory control ratio (RCR) was calculated as the ratio of State III to State IV respiration. Data acquisition and analysis were performed according to standard Oroboros O2k protocols.

### 4.16. Lactate Dehydrogenase (LDH) Assay

Lactate dehydrogenase (LDH) activity in cardiac tissue was measured using a commercial assay kit (Abcam, Cat. No. ab197000; Cambridge, UK) according to the manufacturer’s instructions. Briefly, frozen heart tissue was homogenized in assay buffer and centrifuged at 10,000× *g* for 15 min at 4 °C, and the resulting supernatant was collected for analysis. Samples were incubated with the reaction mixture at 37 °C for 30 min protected from light, and absorbance was measured at 450 nm using a Varioskan LUX Multimode Microplate Reader (Thermo Fisher Scientific, Waltham, MA, USA). LDH activity was determined using an NADH standard curve, normalized to total protein concentration measured by Pierce™ BCA Protein Assay Kit (Thermo Fisher Scientific, Waltham, MA, USA).

### 4.17. Western Blotting

Tissue lysates were prepared using RIPA buffer (Thermo Fisher Scientific, Waltham, MA, USA) supplemented with protease and phosphatase inhibitor cocktail (Thermo Fisher Scientific, Waltham, MA, USA). Protein concentration was determined using the Pierce™ BCA Protein Assay Kit (Thermo Fisher Scientific, Waltham, MA, USA). Protein samples containing 70 μg of protein were mixed with 4× Laemmli loading buffer (Bio-Rad Laboratories, Hercules, CA, USA), denatured at 95 °C for 5 min, and separated on 4–15% TGX Stain-Free gels (Bio-Rad Laboratories, Hercules, CA, USA) at 100 V for 45 min. Proteins were transferred onto PVDF membranes (Bio-Rad Laboratories, Hercules, CA, USA) using the Trans-Blot Turbo Transfer System (Bio-Rad Laboratories, Hercules, CA, USA) at 25 V and 1.0–1.3 A for 3–7 min. Membranes were blocked in 5% non-fat dry milk in Tris-buffered saline containing 0.1% Tween-20 (TBST) for 1 h at room temperature and incubated overnight at 4 °C with primary antibodies diluted in the same blocking buffer against IF1 (bcam, Cat. No. ab110277; 1:500; Cambridge, UK) and vinculin (anta Cruz Biotechnology, Cat. No. sc-25336; 1:500; Dallas, TX, USA). After washing with TBST, membranes were incubated for 1 h at room temperature with HRP-conjugated anti-rabbit IgG (Cell Signaling Technology, Cat. No. 7074; 1:1000; Danvers, MA, USA) or anti-mouse IgG (Cell Signaling Technology, Cat. No. 7076; 1:1000; Danvers, MA, USA). Immunoreactive bands were visualized using enhanced chemiluminescence. Densitometric analysis was performed using Image Lab software (Bio-Rad Laboratories, Hercules, CA, USA), and target protein expression was normalized to vinculin.

### 4.18. Quantitative Real-Time PCR (qPCR) Analysis

Frozen tissue samples were homogenized using the TissueLyser II. Total RNA, including microRNA, was isolated using the miRNeasy Mini Kit (QIAGEN, Hilden, Germany). according to the manufacturer’s instructions. RNA concentration and purity were assessed using a NanoDrop Spectrophotometer (Thermo Fisher Scientific, Waltham, MA, USA), and RNA integrity was confirmed using the TapeStation System (Agilent Technologies, Santa Clara, CA, USA). One-step quantitative real-time PCR (qPCR) was performed using the Power SYBR™ Green RNA-to-CT™ 1-Step Kit (Thermo Fisher Scientific, Waltham, MA, USA) on a LightCycler 480 System (Roche Diagnostics, Basel, Switzerland). Gene-specific primers were synthesized Integrated DNA Technologies (IDT, Coralville, IA, USA). Primer sequences were as follows: IF1 forward, 5′-GAACGATATTTCCGAGCACAG-3′; IF1 reverse, 5′-TGGCGCTCAATTTCTTTCTGC-3′; GAPDH forward, 5′-CATCTTCCAGGAGCGAGACC-3′; and GAPDH reverse, 5′-CCTTCAAGTGGGCCCCG-3′.

### 4.19. Statistical Analysis

Statistical analyses were performed using GraphPad Prism version 10 (GraphPad Software, Boston, MA, USA). Data are presented as mean ± SEM. Comparisons between two groups were performed using unpaired two-tailed Student’s *t*-tests, whereas comparisons among multiple groups were analyzed using one-way or two-way ANOVA followed by appropriate post hoc multiple-comparisons tests. A *p* value < 0.05 was considered statistically significant.

## Data Availability

The raw data supporting the conclusions of this article will be made available by the authors on request.

## Supplementary Materials

The following supporting information can be downloaded at: https://www.mdpi.com/article/10.3390/ijms27146360/s1.
