# Supplementary material for: ATP Synthase Inhibitory Factor-1 Deficiency Attenuates Doxorubicin Cardiotoxicity by Preserving Mitochondrial Structure and Function
Source: Int J Mol Sci. 2026 Jul 17;27(14):6360. doi: 10.3390/ijms27146360 (PMC13409920; doi:10.3390/ijms27146360)
Supplement: Supplementary file 1 [file ijms-27-06360-s001.zip › ijms-4385219-supplementary.pdf]

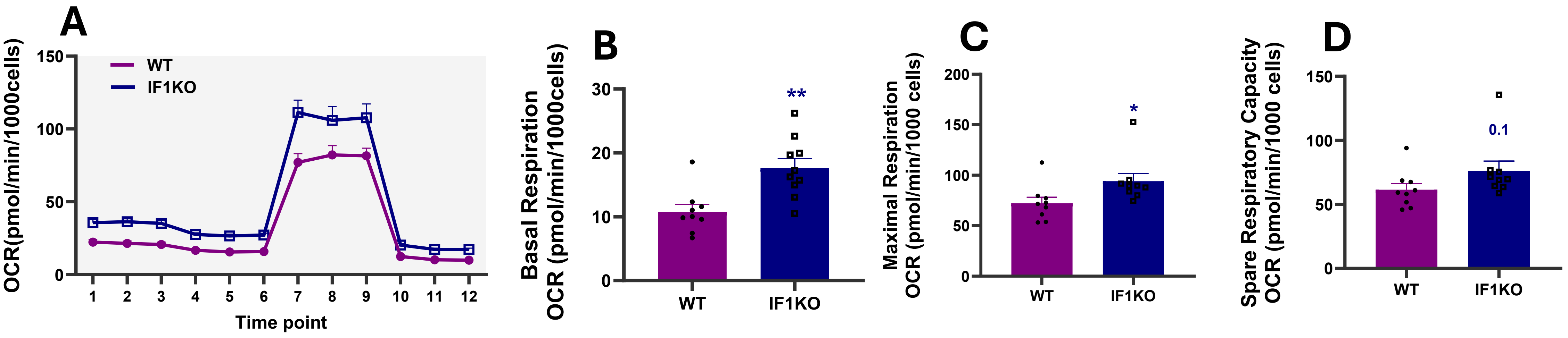


**Supplementary Figure S1: IF1KO enhances mitochondrial respiration at baseline in NCMs.** (A-D) Seahorse XF traces showing mitochondrial respiration in neonatal cardiomyocytes isolated from WT and IF1KO and quantification of basal, maximal and spare respiratory capacity. Statistical analysis was performed using Students' t-test. (*) indicates the comparison between WT and IF1KO. *P < 0.05.

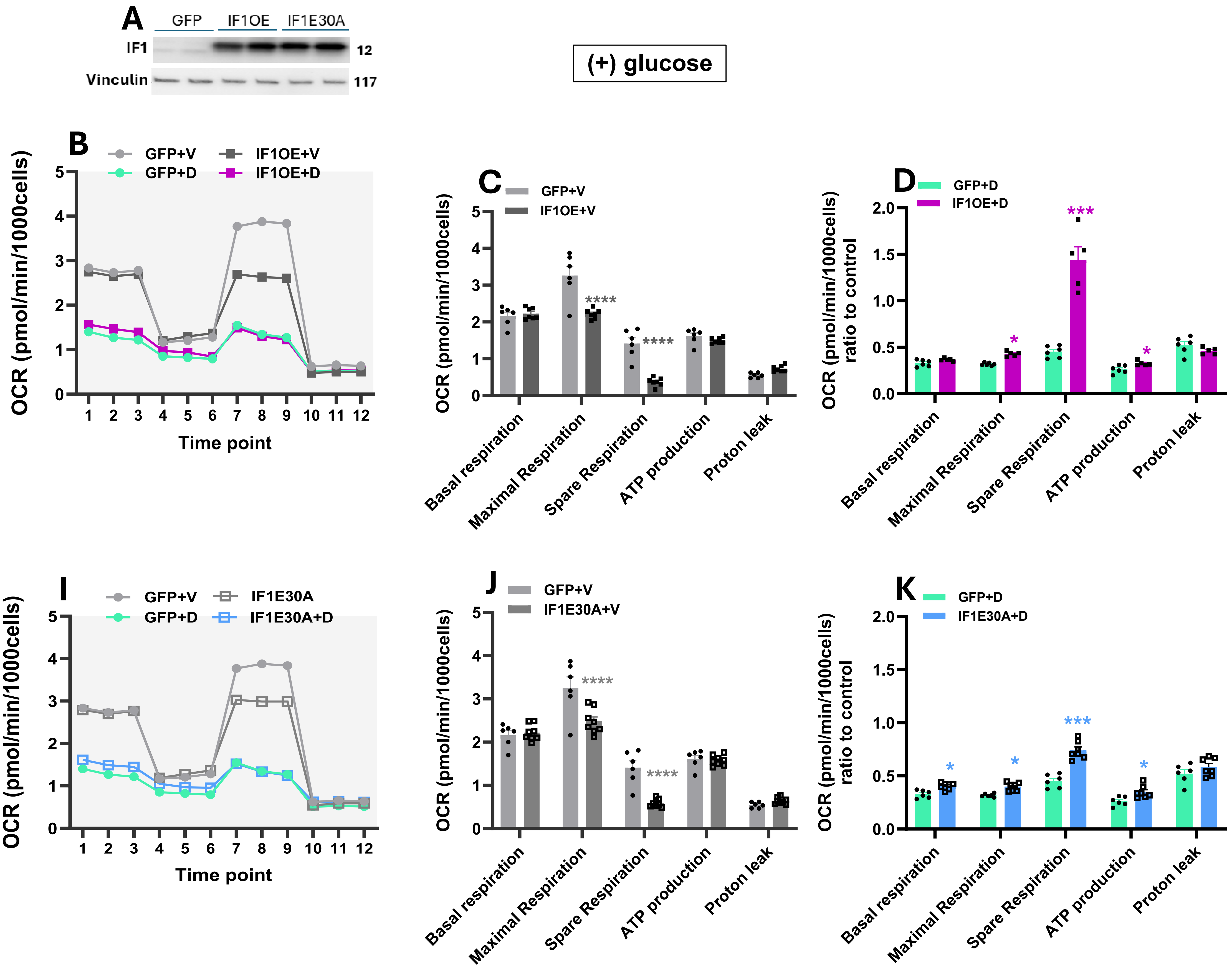


**Supplementary Figure S2: In glucose-supplemented media, both IF1OE and IF1E30A enhance mitochondrial respiration under stress.** (A) Representative Western blot showing IF1 protein expression in GFP-tagged control, IF1OE, and IF1E30A cells 28 h post-transduction. (B) Representative Seahorse XF Mito Stress Test trace illustrating oxygen consumption rate (OCR) profiles of IF1OE and WT cells. (C) Quantification of mitochondrial parameters including basal respiration, maximal respiration, spare respiratory capacity, ATP-linked respiration, and proton leak under steady-state conditions. (D–H) Mitochondrial respiratory parameters under DOX stress, normalized to steady-state levels in IF1OE and WT cells. (I) Representative Seahorse XF Mito Stress Test trace showing OCR profiles of IF1E30A and WT cells under basal conditions. (J) Quantification of mitochondrial parameters including basal respiration, maximal respiration, spare respiratory capacity, ATP-linked respiration, and proton leak under steady-state conditions. (K–O) Mitochondrial parameters following DOX treatment, normalized to their respective steady-state values in IF1E30A and WT cells. Data are presented as mean ± SEM. Statistical analysis was performed using two-way ANOVA followed by Fisher's LSD . (*) indicates comparisons between GFP and IF1OE or IF1E30A groups , *P < 0.05; **P < 0.01; ***P < 0.001; ****P < 0.000.

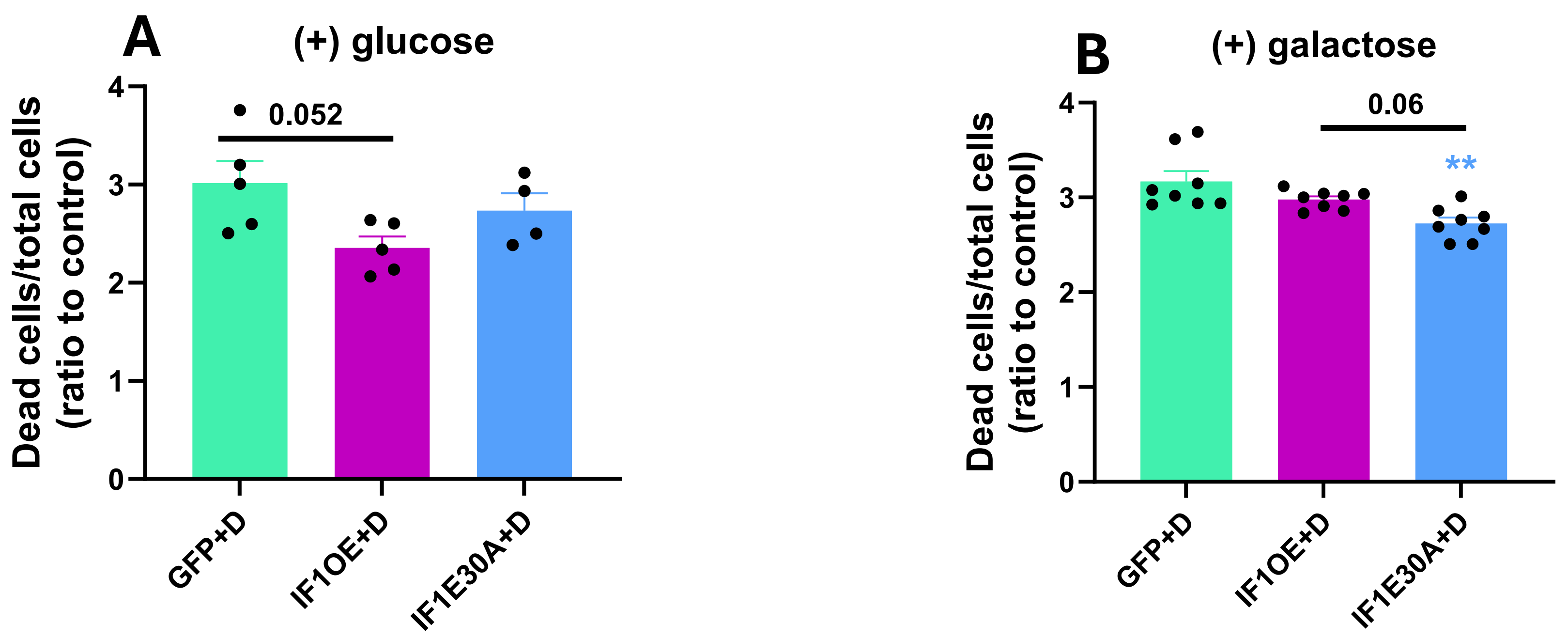


**Supplementary Figure S3. IF1 modulation differentially affects cell death under doxorubicin-induced mitochondrial stress.** (A) Ratio of SYTOX-positive dead cells to total Hoechst-positive cells, normalized to vehicle-treated controls, following 48 h exposure to 1 μM DOX in glucose-containing medium. (B) Ratio of SYTOX-positive dead cells to total Hoechst-positive cells, normalized to vehicle-treated controls, following 48 h exposure to 1 μM DOX in galactose-containing medium. Data were analyzed by one-way ANOVA with Tukey's post hoc test. *indicates comparisons between GFP and IF1OE or IF1E30A groups. **P < 0.01
